# Secondary Metabolites from an Algicolous *Aspergillus versicolor* Strain

**DOI:** 10.3390/md10010131

**Published:** 2012-01-16

**Authors:** Feng-Ping Miao, Xiao-Dong Li, Xiang-Hong Liu, Robert H. Cichewicz, Nai-Yun Ji

**Affiliations:** 1 Yantai Institute of Coastal Zone Research, Chinese Academy of Sciences, Yantai 264003, China; Email: fpmiao@yic.ac.cn (F.-P.M.); imnli@163.com (X.-D.L.); xianghong1127@163.com (X.-H.L.); 2 Natural Products Discovery Group, Department of Chemistry and Biochemistry, University of Oklahoma, Norman, OK 73019, USA; Email: rhcichewicz@ou.edu

**Keywords:** *Sargassum thunbergii*, *Aspergillus versicolor*, asperversin A, 9ξ-*O*-2(2,3-dimethylbut-3-enyl)brevianamide Q

## Abstract

Two new compounds, asperversin A (**1**) and 9ξ-*O*-2(2,3-dimethylbut-3-enyl)brevianamide Q (**2**), and nine known compounds, brevianamide K (**3**), brevianamide M (**4**), aversin (**5**), 6,8-di-*O*-methylnidurufin (**6**), 6,8-di-*O*-methylaverufin (**7**), 6-*O*-methylaverufin (**8**), 5*α*,8*α*-epidioxyergosta-6,22-dien-3*β*-ol (**9**), ergosta-7,22-diene-3*β*,5*α*,6*β*-triol (**10**), and 6*β*-methoxyergosta-7,22-diene-3*β*,5*α*-diol (**11**), were obtained from the culture of *Aspergillus versicolor*, an endophytic fungus isolated from the marine brown alga *Sargassum thunbergii*. The structures of these compounds were established by spectroscopic techniques. Compounds **4**, **7** and **8** exhibited antibacterial activities against *Escherichia coli* and *Staphyloccocus aureus*, and **7** also showed lethality against brine shrimp (*Artemia salina*) with an LC_50_ value of 0.5 μg/mL.

## 1. Introduction

As primary producers, marine algae are faced with a variety of survival stresses, including predation and diseases caused by microorganisms. Algicolous fungi, which are widespread among marine algae, are proposed to play important ecological adaptations for the host that include providing increased resistance against biotic stresses. These protective effects are thought to be mediated by fungal-derived natural products, which makes algicolous fungi and their secondary metabolites a valuable resource for new bioactive compound discovery [1,2].

**Figure 1:**
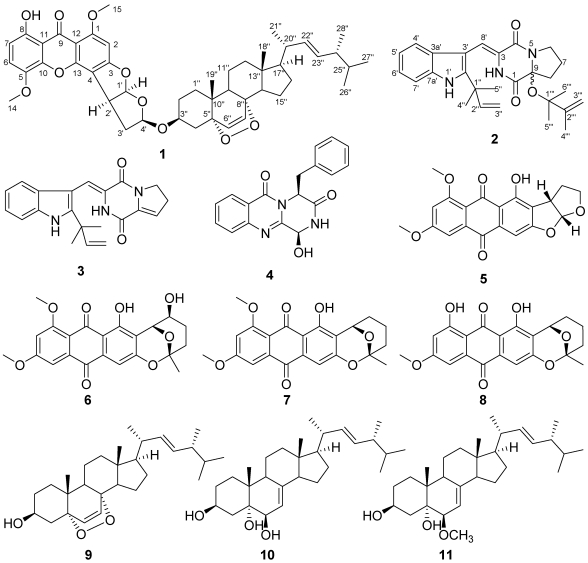
Structures of compounds **1**–**11**.

The brown alga *Sargassum thunbergii* is distributed widely throughout the marine environment surrounding eastern China and it is recognized for its ability to generate large quantities of biomass. In our program focused on the identification of new and bioactive compounds from marine algae and their associated fungi, endophytic fungi from *S. thunbergii* (collected near Pingtan Island) were selected for chemical investigations. In the course of these studies, a strain of *Aspergillus versicolor* (strain designation pt20) was isolated from the inner tissue of *S. thunbergii*. Our chemical investigation of this strain resulted in the identification of two new compounds, asperversin A (**1**) and 9ξ-*O*-2(2,3-dimethylbut-3-enyl)brevianamide Q (**2**), as well as nine known compounds that included brevianamide K (**3**) [3], brevianamide M (**4**) [3], aversin (**5**) [4], 6,8-di-*O*-methylnidurufin (**6**) [5], 6,8-di-*O*-methylaverufin (**7**) [6], 6-*O*-methylaverufin (**8**) [7], 5*α*,8*α*-epidioxyergosta-6,22-dien-3*β*-ol (**9**) [8], ergosta-7,22-diene-3*β*,5*α*,6*β*-triol (**10**) [9], and 6*β*-methoxyergosta-7,22-diene-3*β*,5*α*-diol (**11**) [9]. This paper describes the isolation, structure determination, and bioactivity of compounds **1**–**11** (Figure 1).

## 2. Results and Discussion

Compound **1** was obtained as yellow crystals from CHCl_3_. The molecular formula was determined as C_47_H_58_O_10_ on the basis of HREIMS with the [M]^+^ peak at *m/z* 782.4024 (calcd. for C_47_H_58_O_10_, 782.4030), indicating nineteen degrees of unsaturation. The ^1^H-NMR spectrum (Table 1) displayed two tertiary methyl singlets, four secondary methyl doublets, two oxygenated methyl singlets, one multiplet and two doublets assignable to three oxygenated methines, four doublets ascribed to two mutually coupled olefinic protons and two mutually coupled aromatic protons, two double doublets attributed to two mutually coupled olefinic protons, and one singlet characteristic of an aromatic proton. The ^13^C NMR spectrum (Table 1) along with the DEPT and HSQC experiments revealed the presence of eight methyl groups, eight methylenes, seventeen methines, and fourteen quaternary carbon atoms. Upon further inspection of the NMR data, it was realized that approximately half of the chemical shifts in **1** were superimposeable with 5*α*,8*α*-epidioxyergosta-6,22-dien-3*β*-ol (**9**), which we also isolated from the same fungal extract [8]. This enabled us to focus on the remaining chemical shifts in **1**, which we were able to deduce had remarkable similarities to the natural product 5-methoxysterigmatocystin [4]. However, several key differences remained between our NMR data and those reported for 5-methoxysterigmatocystin. Namely, our metabolite **1** exhibited new resonances for a methylene and an oxygenated methine, but lacked two olefinic methines. Therefore, we deduced that this portion of the molecule represented a 3',4'-dihydro-5-methoxysterigmatocystin residue, which was verified by the ^1^H–^1^H COSY correlations between H-6/H-7, H-1'/H-2', H-2'/H-3', H-3'/H-4' and HMBC correlations from H-2 to C-1, C-3, C-4, and C-12, from H-6 to C-5, C-8, and C-10, from H-7 to C-5, C-8, and C-11, from H-14 to C-5, from H-15 to C-1, from H-1' to C-3, C-4, C-2', C-3', and C-4', from H-2' to C-3, C-4, C-13, C-1', C-3', and C-4', and from H-4' to C-1', C-2', and C-3'. The linkage between the two portions of compound **1** was established by HMBC correlations from H-4' to C-3" and from H-3" to C-4'. The relative configuration of **1** was confirmed by analysis of NOESY spectrum. Based on biogenetic considerations, the configurations of steroid moiety should be the same as those of 5*α*,8*α*-epidioxyergosta-6,22-dien-3*β*-ol (**9**) [8], which were confirmed by the observed NOESY correlations between H-6"/H-19", H-7"/H-18", H-18"/H-20", H-2"a/H-19", H-2"b/H-3". H-3"and H-4' were located on the same side by the NOESY correlation between H-3"/H-4', while H-4', H-1', and H-2' were positioned on the same face based on the NOESY correlations of H-1' with H-2' and H-4'. We have given this new molecule from *A. versicolor* pt20 the trivial name asperversin A.

**Table 1:** ^1^H and ^13^C NMR data for compound **1** (500 MHz for ^1^H and 125 MHz for ^13^C, CDCl_3_).

Position	δ_H_ (*J* in Hz)	δ_C_, mult.	Position	δ_H_(*J* in Hz)	δ_C_, mult.
1		163.3, C	6"	6.11, d (8.5)	135.3, CH
2	6.34, s	90.1, CH	7"	6.44, d (8.5)	130.7, CH
3		165.3, C	8"		79.4, C
4		108.2, C	9"	1.44, m	51.1, CH
5		139.4, C	10"		37.1, C
6	7.18, d (9.0)	120.4, CH	11"a	1.36, m	20.6, CH_2_
7	6.68, d (9.0)	109.3, CH	11"b	1.56, m	
8		155.3, C	12"a	1.20, m	39.3, CH_2_
9		181.6, C	12"b	1.92, m	
10		144.9, C	13"		44.5, C
11		109.6, C	14"	1.52, m	51.6, CH
12		105.7, C	15"a	1.16, m	23.3, CH_2_
13		153.9, C	15"b	1.45, m	
14	3.91, s	57.8, CH_3_	16"a	1.33, m	28.6, CH_2_
15	3.99, s	56.8, CH_3_	16"b	1.73, m	
1'	6.51, d (6.0)	113.5, CH	17"	1.20, m	56.2, CH
2'	4.21, dd (9.2, 6.0)	43.0, CH	18"	0.78, s	12.8, CH_3_
3'a	2.34, ddd (13.2, 9.2, 4.9)	36.9, CH_2_	19"	0.72, s	18.0, CH_3_
3'a	2.47, d (13.2)		20"	2.00, m	39.7, CH
4'	5.39, d (4.9)	104.2, CH	21"	0.98, d (6.6)	20.9, CH_3_
1"a	1.57, m	34.6, CH_2_	22"	5.13, dd (15.3, 8.3)	135.2, CH
1"b	1.86, m		23"	5.21, dd (15.3, 7.6)	132.3, CH
2"a	1.13, m	27.6, CH_2_	24"	1.84, m	42.8, CH
2"b	1.73, m		25"	1.45, m	33.1, CH
3"	3.79, m	72.1, CH	26"	0.81, d (6.8)	19.6, CH_3_
4"a	1.54, m	33.6, CH_2_	27"	0.83, d (6.8)	20.0, CH_3_
4"b	1.91, m		28"	0.90, d (6.8)	17.6, CH_3_
5"		81.8, C	OH	12.73, s	

Compound **2** was obtained as colorless crystals from CHCl_3_. The molecular formula was established to be C_27_H_33_N_3_O_3_ based on HREIMS (*m/z* 447.2507 [M]^+^, calcd. for C_27_H_33_N_3_O_3_, 447.2522), requiring thirteen degrees of unsaturation. The ^1^H NMR spectrum (Table 2) of **2** exhibited five methyl singlets, two singlets characteristic of terminal olefinic protons, one double doublet and two doublets attributed to a terminal vinyl group, two doublets and two double doublets ascribed to an ortho-substituted phenyl group, and two broad singlets assigned to two presumably exchangeable protons. The ^13^C NMR spectrum (Table 2) exhibited fifteen resonances, which were identified as five methyls, five methylenes, six methines, and eleven quaternary carbons by the DEPT and HSQC experiments. The HMBC correlations from H-3"' to C-1"', C-2"', and C-4"', from H-4"' to C-1"', C-2"', and C-3"', from H-5"' to C-1"', C-2"', and C-6"', and from H-6"' to C-1"', C-2"', and C-5"' established the presence of structural unit CH_2_=CH(CH_3_)–C(CH_3_)_2_–. The remaining NMR resonances were similar to those reported for brevianamide Q [10]. The major exception was the significant downfield shift of C-9 (δ_C_ 94.3) due to it being the site of an ether linkage to the new CH_2_=CH(CH_3_)–C(CH_3_)_2_– group. The structure of the remaining portion of the compound was confirmed based on ^1^H–^1^H COSY correlations between H-6/H-7, H-7/H-8, H-4'/H-5', H-5'/H-6', H-6'/H-7', H-2"/H-3", as well as HMBC correlations from H-1 to C-4, from H-8 to C-1 and C-9, from H-1' to C-3' and C-3a', from H-4' to C-3', C-6', and C-7a', from H-7' to C-3a' and C-5', from H-8' to C-4 and C-2', and from H-4" and H-5" to C-2', C-1", and C-2". The above evidence established the structure of **2**, named 9ξ-*O*-2(2,3-dimethylbut-3-enyl)brevianamide Q.

**Table 2:** ^1^H and ^13^C NMR data for compound **2** (500 MHz for ^1^H and 125 MHz for ^13^C, CDCl_3_).

Position	δ_H_ (*J* in Hz)	δ_C_, mult.	Position	δ_H_(*J* in Hz)	δ_C_, mult.
1		162.3, C	6'	7.19, dd (7.5, 8.0)	122.3, CH
2	7.48 br, s		7'	7.35, d (8.0)	111.1, CH
3		126.2, C	7a'		134.2, C
4		159.4, C	8'	7.29, s	111.6, CH
5			1"		39.3, C
6a	3.75, m	45.2, CH_2_	2"	6.08, dd (17.4, 10.6)	144.3, CH
6b	3.95, m		3"a	5.21, d (17.4)	113.4, CH_2_
7a	2.00, m	19.8, CH_2_	3"b	5.24, d (10.6)	
7b	2.10, m		4"	1.54, s	27.3, CH_3_
8a	2.25, m	33.5, CH_2_	5"	1.54, s	27.7, CH_3_
8b	2.37, m		1"'		84.6, C
9		94.3, C	2"'		148.1, C
1'	8.29 br, s		3"'a	4.89, s	111.7, CH_2_
2'		144.1, C	3"'b	4.96, s	
3'		103.5, C	4"'	1.83, s	18.5, CH_3_
3a'		126.2, C	5"'	1.40, s	24.0, CH_3_
4'	7.47, d (7.5)	119.9, CH	6"'	1.32, s	25.0, CH_3_
5'	7.13, dd (7.5, 7.5)	120.9, CH			

In addition to these two new compounds, we also isolated several known compounds including brevianamide K (**3**) [3], brevianamide M (**4**) [3], aversin (**5**) [4], 6,8-di-*O*-methylnidurufin (**6**) [5], 6,8-di-*O*-methylaverufin (**7**) [6], 6-*O*-methylaverufin (**8**) [7], 5*α*,8*α*-epidioxyergosta-6,22-dien-3*β*-ol (**9**) [8], ergosta-7,22-diene-3*β*,5*α*,6*β*-triol (**10**) [9], and 6*β*-methoxyergosta-7,22-diene-3*β*,5*α*-diol (**11**) [9]. The structures of these metabolites were confirmed by comparisons of their respective spectroscopic data with those reported earlier.

**Table 3:** Antibacterial activities at 30 μg/disk and toxicities against brine shrimp at 100 μg/mL of **1**–**8**.

Compounds	Inhibition Zone (mm)		Lethal Rates (%)
	*Escherichia coli*	*Staphylococcus aureus*	*Artemia salina*
**1**	7	7	1.8
**2**	7	7	43.2
**3**	7	7	30.9
**4**	11	10	47.6
**5**	6	6	17.5
**6**	7	7	29.1
**7**	10	10	100.0
**8**	10	10	38.5
chloramphenicol	32	31	

Compounds **1**–**8** were tested for biological activities against several target organisms including bacteria, fungi, and brine shrimp. Antibacterial activity was assessed by disk diffusion assay against *Escherichia coli* and *Staphylococcus aureus* at a concentration of 30 μg/disk. Compounds **4**, **7**, and **8** were found to exhibit modest inhibitory activity against these bacterial strains (Table 3). None of the compounds inhibited the fungal species *Colletotrichum lagenarium* or *Fusarium oxysporium* at 30 μg/disk in the disk diffusion assay [11]. Interestingly, compound **7** exhibited significant toxicity toward brine shrimp with an LC_50_ value of 0.5 μg/mL [12].

## 3. Experimental Section

### 3.1. General

NMR spectra were recorded in CDCl_3_ at 500 and 125 MHz for ^1^H and ^13^C, respectively, on a Bruker Avance III 500 NMR spectrometer using TMS as internal standard. High resolution mass data were acquired on Autospec Premier P776 mass spectrometer with an EI source. IR spectra were obtained on a JASCO FT/IR-4100 Fourier Transform InfraRed spectrometer. UV spectrum was measured on a TU-1810 Spectrophotometer. HPLC separation was carried out on an Elite HPLC system (P270 pump, UV230+ detector, Dalian Elite Analytical Instruments Co., Ltd., Dalian, China) using an Eclipse XDB-C18 (5 μm, 9.4 × 250 mm) column. Column chromatography was performed with silica gel (100–200 and 200–300 mesh, Qingdao Haiyang Chemical Co., Qingdao, China) and Sephadex LH-20 (Pharmacia). Precoated silica gel plates (GF-254, Qingdao Haiyang Chemical Co., Qingdao, China) were used for preparative TLC purification. All solvents were of analytical grade.

### 3.2. Microorganism and Fermentation

The endophytic fungus *A. versicolor* pt20 was isolated from a fresh, surface-sterilized tissue sample of the marine brown alga *S. thunbergii*, which was collected from Pingtan Island, China. The fungus was identified based on morphological and molecular taxonomic methods by one of the authors (F.-P.M.). A voucher sample has been preserved in Bio-Resource Laboratory of Yantai Institute of Coastal Zone Research, Chinese Academy of Sciences. The initial cultures were maintained on the potato dextrose agar plates. Pieces of mycelia were cut into small segments and aseptically inoculated into 1000 mL Erlenmeyer flasks containing 300 mL potato dextrose broth (PDB) culture media. The static fermentation was carried out for 30 days at room temperature (25 °C).

### 3.3. Extraction and Isolation

The culture broth (15 L) was extracted with EtOAc to yield 3.6 g gum after removal of the solvent by evaporation (40 °C) at reduced pressure. The dried and powdered mycelia (162.2 g) were extracted with a mixture of CHCl_3_ and MeOH (1:1, v/v), concentrated, and partitioned between H_2_O and EtOAc to give 29.8 g gum. Since the TLC profiles of the two extracts were nearly identical, they were combined before further separation. The total EtOAc-soluble fraction (33.4 g) was subjected to silica gel column chromatography (CC, petroleum ether (PE)/EtOAc gradient) to afford 16 fractions (Fr. 1–16), monitored by TLC. Fr. 10 eluted with PE/EtOAc (5:1) and was further purified by CC on Sephadex LH-20 (CHCl_3_/MeOH, 1:1) to yield three sub-fractions, 10-1, 10-2, and 10-3. Sub-fraction 10-1 was further purified by silica gel CC (PE/EtOAc, 5:1) and HPLC (MeOH/H_2_O, 85%) to give **9** (3.0 mg). Sub-fraction 10-2 was further purified by silica gel CC (PE/EtOAc, 4:1) and preparative TLC (CHCl_3_/EtOAc, 2:1) to afford **7** (13.0 mg). Sub-fraction 10-3 was further purified by silica gel CC (CHCl_3_/EtOAc, 4:1) and preparative TLC (CHCl_3_/EtOAc, 3:2) to produce **1** (8.3 mg). Fr. 11 eluted with PE/EtOAc (2:1) and was further purified by CC on Sephadex LH-20 (CHCl_3_/MeOH, 1:1) to afford **5** (27.1 mg). Fr. 12 eluted with PE/EtOAc (2:1) and was further purified by CC on Sephadex LH-20 (CHCl_3_/MeOH, 1:1) and preparative TLC (CHCl_3_/EtOAc, 3:2) to yield **2** (2.0 mg). Fr. 13 eluted with PE/EtOAc (1:1) and was further purified by CC on Sephadex LH-20 (CHCl_3_/MeOH, 1:1) to produce two sub-fractions, 13-1 and 13-2. Sub-fraction 13-1 was further purified by silica gel (PE/EtOAc, 2:1) and preparative TLC (CHCl_3_/EtOAc, 2:1) to give **3** (4.3 mg). Sub-fraction 13-2 was further purified by CC on silica gel (PE/EtOAc, 2:1–1:1) and HPLC (MeOH /H_2_O, 85%) to afford **11** (4.4 mg). Fr. 14 eluted with PE/EtOAc (1:1) and was further purified by CC on Sephadex LH-20 (CHCl_3_/MeOH, 1:1) and silica gel (PE/EtOAc, 1:1) and preparative TLC (CHCl_3_/EtOAc, 1:1) to yield **6** (2.0 mg). Fr. 15 eluted with EtOAc and was further purified by CC on Sephadex LH-20 (CHCl_3_/MeOH, 1:1) to yield three sub-fractions, 15-1, 15-2, and 15-3. Sub-fraction 15-1 was further purified by CC on silica gel (PE/EtOAc, 1:2) and preparative TLC (PE/EtOAc, 1:2) to afford **8** (10.2 mg). Sub-fraction 15-2 was further purified by CC on silica gel (CHCl_3_/EtOAc, 2:3) and preparative TLC (CHCl_3_/EtOAc, 2:3) to produce **4** (29.0 mg). Sub-fraction 15-3 was further purified by HPLC (MeOH /H_2_O, 85%) and preparative TLC (EtOAc) to give **10** (3.0 mg).

*Asperversin A* (**1**): Yellow crystals; m.p. 273–275 °C; [α]^25^_D_ −309.7 (*c* 0.12, CHCl_3_); UV (CHCl_3_) λ_max_ (log ε) 248 (4.51), 330 (4.14) nm. IR (KBr) *ν*_max_ 3448, 2927, 2866, 1631, 1581, 1485, 1369, 1234, 972 cm^−1^. ^1^H and ^13^C NMR data, see Table 1. HREIMS *m/z* 782.4024 [M]^+^, calcd. for C_47_H_58_O_10_, 782.4030.

9ξ-*O*-2(2,3-dimethylbut-3-enyl)brevianamide Q (**2**): Colorless crystals; m.p. 89–92 °C; [α]^24^_D_ −16.0 (*c* 0.11, CHCl_3_); UV (CHCl_3_) λ_max_ (log ε) 238 (3.61), 334 (3.28) nm. IR (KBr) *ν*_max_ 3340, 2927, 2858, 1693, 1624, 1427, 1385, 1238, 1149, 910, 748 cm^−1^. ^1^H and ^13^C NMR data, see Table 2. HREIMS *m/z* 447.2507 [M]^+^, calcd. for C_27_H_33_N_3_O_3_, 447.2522.

### 3.4. Antimicrobial Assay

Antibacterial and antifungal activities were assayed as described previously [11].

### 3.5. Brine Shrimp Lethality Assay

Brine shrimp (*Artemia salina*) lethality assay procedure followed the micro-well plate method described by Solis *et al* with some modifications [12]. Briefly, brine shrimp eggs were left to hatch in sea water for 48 hours at 28 °C under natural light. For brine shrimp lethality testing, compounds were dissolved in DMSO prior to preparing serial dilutions in 200 µL volume of sea water prepared in 96 well microplates. A well containing DMSO without compounds added was used as a positive control. Approximately, 10 brine shrimp were placed in a well with a volume of 200 µL sea water for lethality testing. Brine shrimp lethality was observed after 24 hours of cultivation under continuous light. Dead shrimp were identified with the aid of a handheld magnifying lens. 

## 4. Conclusions

In summary, two new (**1** and **2**) and nine known (**3**–**11**) secondary metabolites were purified from the algicolous fungus *A. versicolor* pt20. To the best of our knowledge, compound **1** represented the first described example of a steroid-xanthone heterodimer. Compunds **4**, **7**, and **8** were more active against *E. coli* and *S. aureus*, and **7** also showed strong toxicity against brine shrimp.

## Supplementary Files

Supplementary File 1: PDF-Document (PDF, 1791 KB)
