# Supplementary material for: Secondary Metabolites from an Algicolous Aspergillus versicolor Strain
Source: Mar Drugs. 2012 Jan 16;10(1):131–9. doi: 10.3390/md10010131 (PMC3280527; doi:10.3390/md10010131)
Supplement: Supplementary File 1: — PDF-Document (PDF, 1791 KB) [file marinedrugs-10-00131-s001.pdf]

Supporting information

Secondary Metabolites from an Algicolous Endophytic *Aspergillus versicolor* Strain

Feng-Ping Miao, Xiao-Dong Li, Xiang-Hong Liu and Nai-Yun Ji*
Yantai Institute of Coastal Zone Research, Chinese Academy of Sciences, Yantai 264003, China;
E-Mails: fpmiao@yic.ac.cn (F.-P.M.); imnli@163.com (X.-D.L.); xianghong1127@163.com (X.-H.L.)

* Author to whom correspondence should be addressed; E-Mail: nyji@yic.ac.cn; Tel.: +86 535 2109176; Fax: +86 535 2109000

List of supporting information

S2, ^{1}H NMR spectrum of compound **1**;

S3, ^{13}C NMR and DEPT spectra of compound **1**;

S4, HSQC spectrum of compound **1**;

S5, HMBC spectrum of compound **1**;

S6, ^{1}H-^{1}H COSY spectrum of compound **1**;

S7, NOESY spectrum of compound **1**;

S8, HREIMS spectrum of compound **1**;

S9, ^{1}H NMR spectrum of compound **2**;

S10, ^{13}C NMR and DEPT spectra of compound **2**;

S11, HSQC spectrum of compound **2**;

S12, HMBC spectrum of compound **2**;

S13, ^{1}H-^{1}H COSY spectrum of compound **2**;

S14, NOESY spectrum of compound **2**;

S15, HREIMS spectrum of compound **2**;

pt20-12

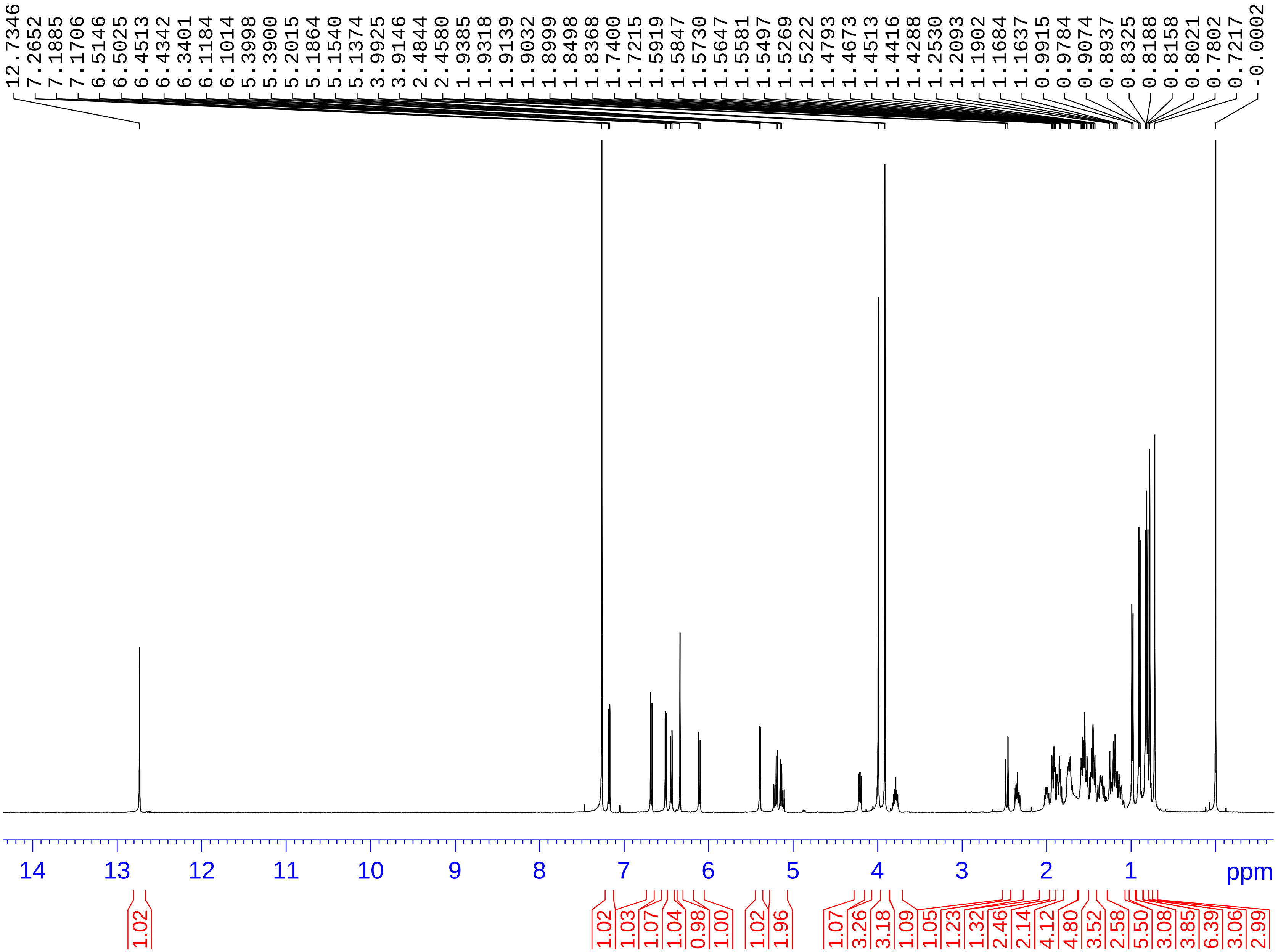

NAME	110808
EXPNO	5
PROCNO	1
Date_	20110809
Time	9.09
INSTRUM	spect
PROBHD	5 mm PABBO BB-
PULPROG	zg30
TD	65536
SOLVENT	CDCl3
NS	16
DS	0
SWH	10330.578 Hz
FIDRES	0.157632 Hz
AQ	3.1719923 sec
RG	128
DW	48.400 usec
DE	6.50 usec
TE	293.5 K
D1	1.00000000 sec
TD0	1

======== CHANNEL f1 ========

NUC1	1H
P1	12.40 usec
PL1	0.35 dB
PL1W	19.76737595 W
SFO1	500.1330885 MHz
SI	32768
SF	500.1300112 MHz
WDW	EM
SSB	0
LB	0.30 Hz
GB	0
PC	1.00

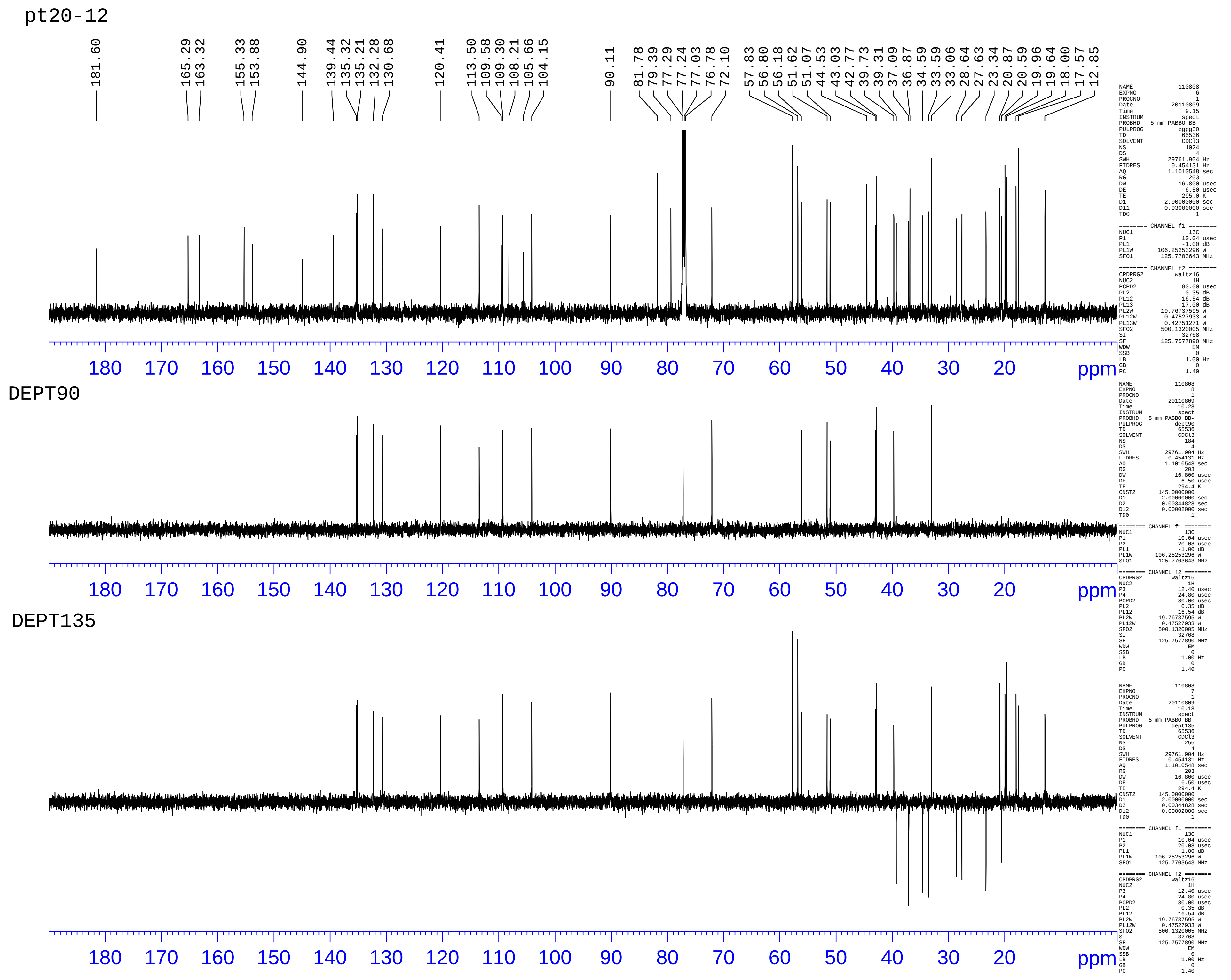
pt20-12
181.60
165.29
163.32
155.33
153.88
144.90
139.44
135.32
135.21
132.28
130.68
120.41
113.50
109.58
109.30
108.21
105.66
104.15
90.11
81.78
79.39
77.29
77.24
77.03
76.78
72.10
57.83
56.80
56.18
51.62
51.07
44.53
43.03
42.77
39.73
39.31
37.09
36.87
34.59
33.59
33.06
28.64
27.63
23.34
20.87
20.59
19.96
19.64
18.00
17.57
12.85
180 170 160 150 140 130 120 110 100 90 80 70 60 50 40 30 20 ppm
DEPT90
180 170 160 150 140 130 120 110 100 90 80 70 60 50 40 30 20 ppm
DEPT135
180 170 160 150 140 130 120 110 100 90 80 70 60 50 40 30 20 ppm

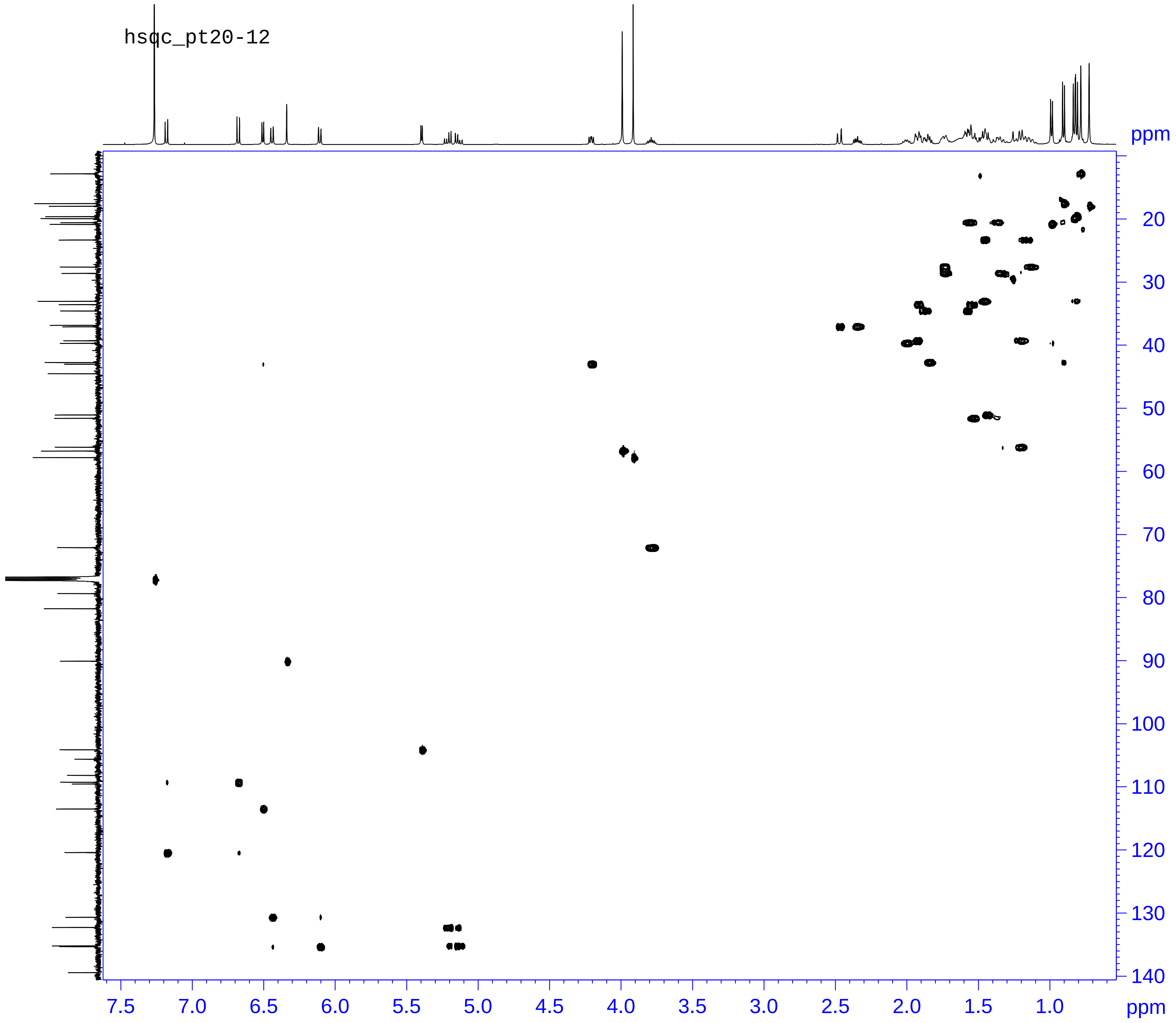

NAME 110812
EXPNO 2
PROCNO 1
Date_ 20110812
Time 9.51
INSTRUM spect
PROBHD 5 mm PABBO BB-
PULPROG hsqcetgpsisp2
TD 2048
SOLVENT CDCl3
NS 4
DS 16
SWH 5000.000 Hz
FIDRES 2.441406 Hz
AQ 0.2048500 sec
RG 203
DW 100.000 usec
DE 6.50 usec
TE 298.5 K
CNST2 145.0000000
D0 0.00000300 sec
D1 2.00000000 sec
D4 0.00172414 sec
D11 0.03000000 sec
D16 0.00020000 sec
D24 0.00086207 sec
IN0 0.00002340 sec
ZGOPTNS

======== CHANNEL f1 ========
NUC1 1H
P1 12.40 usec
P2 24.80 usec
P28 0.00 usec
PL1 0.35 dB
PL1W 19.76737595 W
SFO1 500.1322506 MHz

======== CHANNEL f2 ========
CPDPRG2 garp
NUC2 13C
P3 12.40 usec
P4 24.80 usec
P14 500.00 usec
PCPD2 70.00 usec
PL0 120.00 dB
PL2 0.35 dB
PL12 15.38 dB
PL0W 0.00000000 W
PL2W 77.86446381 W
PL12W 2.44534016 W
SFO2 125.7678496 MHz
SP3 6.64 dB
SPNAM3 Crp60,0.5,20.1
SPOAL3 0.500
SPOFFS3 0.00 Hz

====== GRADIENT CHANNEL =====
GPNAM1 SINE.100
GPNAM2 SINE.100
GPNAM3 SINE.100
GPNAM4 SINE.100
GPZ1 80.00 %
GPZ2 20.10 %
GPZ3 11.00 %
GPZ4 -5.00 %
P16 1000.00 usec
P19 600.00 usec
ND0 2
TD 256
SFO1 125.7678 MHz
FIDRES 83.517715 Hz
SW 170.000 ppm
FnMODE Echo-Antiecho
SI 1024
SF 500.1300120 MHz
WDW QSINE
SSB 2
LB 0.00 Hz
GB 0
PC 1.40
SI 1024
MC2 echo-antiecho
SF 125.7577790 MHz
WDW QSINE
SSB 2
LB 0.00 Hz
GB 0

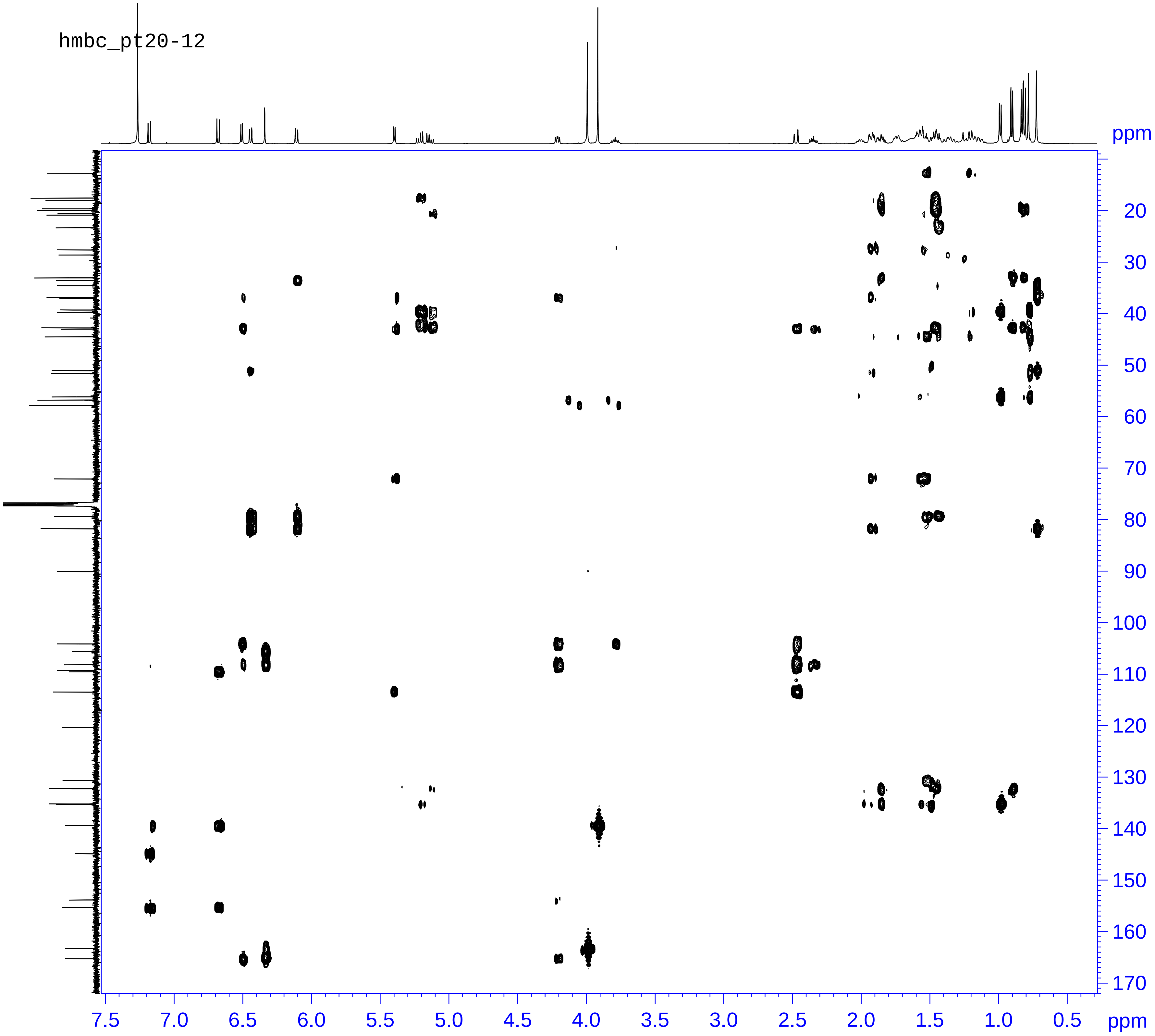

NAME	110812
EXPNO	3
PROCNO	1
Date_	20110812
Time	10.30
INSTRUM	spect
PROBHD	5 mm PABBO BB-
PULPROG	hmbcetgpl2nd
TD	4096
SOLVENT	CDCl3
NS	8
DS	16
SWH	7002.801 Hz
FIDRES	1.709668 Hz
AQ	0.2925044 sec
RG	203
DW	71.400 usec
DE	6.50 usec
TE	298.0 K
CNST6	120.0000000
CNST7	180.0000000
CNST13	7.0000000
CNST30	0.5981173
D0	0.00000300 sec
D1	1.50000000 sec
D6	0.07142857 sec
D16	0.00020000 sec
IN0	0.00001990 sec

======== CHANNEL f1 ========

NUC1	1H
P1	12.40 usec
P2	24.80 usec
PL1	0.35 dB
PL1W	19.76737595 W
SFO1	500.1330008 MHz

======== CHANNEL f2 ========

NUC2	13C
P3	12.40 usec
P24	2000.00 usec
PL2	0.35 dB
PL2W	77.86446381 W
SFO2	125.7697360 MHz
SP7	6.64 dB
SPNAM7	Crp60comp.4
SPOAL7	0.500
SPOFFS7	0.00 Hz

====== GRADIENT CHANNEL =====

GPNAM1	SINE.100
GPNAM3	SINE.100
GPNAM4	SINE.100
GPNAM5	SINE.100
GPZ1	80.00 %
GPZ3	15.00 %
GPZ4	-10.00 %
GPZ5	-5.00 %
P16	1000.00 usec
ND0	2
TD	256
SFO1	125.7697 MHz
FIDRES	98.257607 Hz
SW	200.000 ppm
FnMODE	Echo-Antiecho
SI	1024
SF	500.1300120 MHz
WDW	SINE
SSB	0
LB	0.00 Hz
GB	0
PC	1.40
SI	1024
MC2	echo-antiecho
SF	125.7577790 MHz
WDW	SINE
SSB	0
LB	0.00 Hz
GB	0

cosy_pt20-12

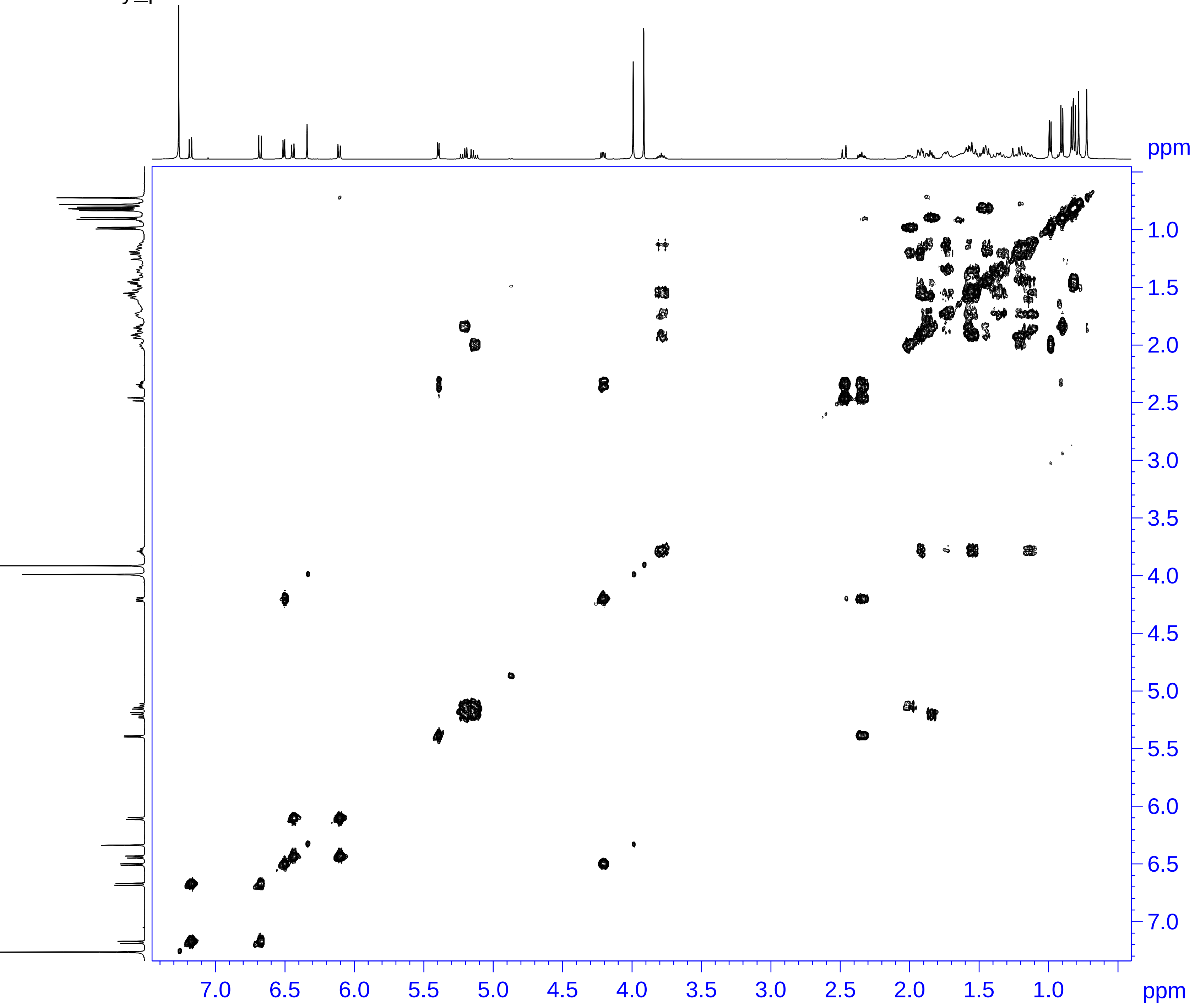

NAME	110812	
EXPNO	1	
PROCNO	1	
Date_	20110812	
Time	9.30	
INSTRUM	spect	
PROBHD	5 mm PABBO BB-	
PULPROG	cosygpmfqf	
TD	2048	
SOLVENT	CDCl3	
NS	2	
DS	16	
SWH	5000.000	Hz
FIDRES	2.441406	Hz
AQ	0.2048500	sec
RG	203	
DW	100.000	usec
DE	6.50	usec
TE	298.2	K
D0	0.00000300	sec
D1	2.00000000	sec
D13	0.00000400	sec
D16	0.00020000	sec
IN0	0.00019995	sec
======== CHANNEL f1 ========		
NUC1	1H	
P1	12.40	usec
PL1	0.35	dB
PL1W	19.76737595	W
SFO1	500.1322506	MHz
====== GRADIENT CHANNEL =====		
GPNAM1	SINE.100	
GPNAM2	SINE.100	
GPNAM3	SINE.100	
GPZ1	16.00	%
GPZ2	12.00	%
GPZ3	40.00	%
P16	1000.00	usec
ND0	1	
TD	256	
SFO1	500.1323	MHz
FIDRES	19.536415	Hz
SW	10.000	ppm
FnMODE	QF	
SI	1024	
SF	500.1300129	MHz
WDW	SINE	
SSB	0	
LB	0.00	Hz
GB	0	
PC	1.40	
SI	1024	
MC2	QF	
SF	500.1300129	MHz
WDW	SINE	
SSB	0	
LB	0.00	Hz
GB	0	

noesy_pt20-12

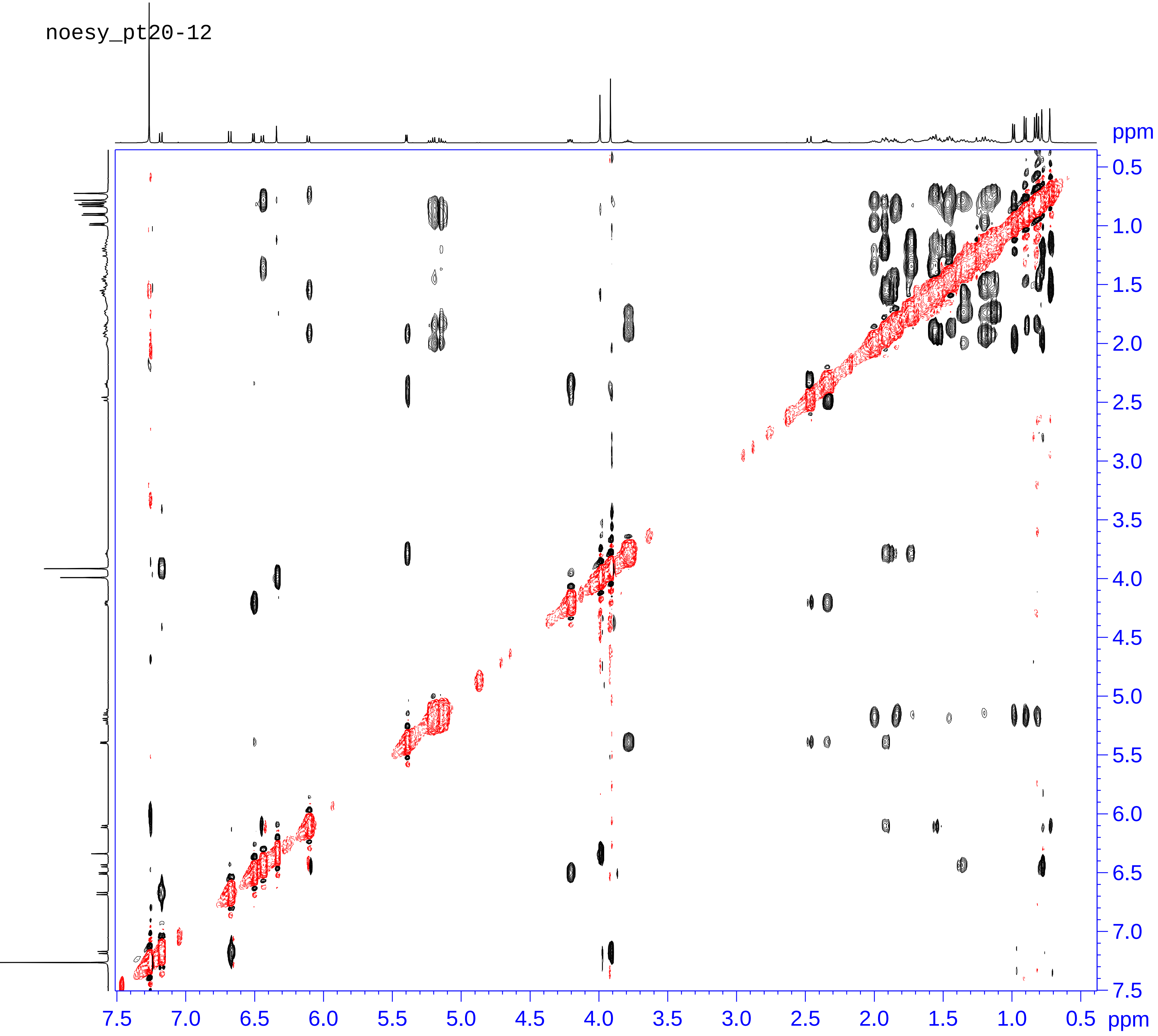

NAME	110812	
EXPNO	4	
PROCNO	1	
Date_	20110812	
Time	11.34	
INSTRUM	spect	
PROBHD	5 mm PABBO BB-	
PULPROG	noesygpph	
TD	2048	
SOLVENT	CDCl3	
NS	4	
DS	8	
SWH	7002.801	Hz
FIDRES	3.419337	Hz
AQ	0.1462772	sec
RG	203	
DW	71.400	usec
DE	6.50	usec
TE	298.2	K
D0	0.00005562	sec
D1	2.00000000	sec
D8	0.69999999	sec
D16	0.00020000	sec
IN0	0.00014280	sec
======== CHANNEL f1 ========		
NUC1	1H	
P1	12.40	usec
P2	24.80	usec
PL1	0.35	dB
PL1W	19.76737595	W
SFO1	500.1330008	MHz
====== GRADIENT CHANNEL =====		
GPNAM1	SINE.100	
GPNAM2	SINE.100	
GPZ1	40.00	%
GPZ2	-40.00	%
P16	1000.00	usec
ND0	1	
TD	256	
SFO1	500.133	MHz
FIDRES	27.351023	Hz
SW	14.000	ppm
FnMODE	States-TPPI	
SI	1024	
SF	500.1300120	MHz
WDW	QSINE	
SSB	2	
LB	0.00	Hz
GB	0	
PC	1.00	
SI	1024	
MC2	States-TPPI	
SF	500.1300120	MHz
WDW	QSINE	
SSB	2	
LB	0.00	Hz
GB	0	

Elemental Composition Report

Single Mass Analysis (displaying only valid results)

Tolerance = 10.0 PPM / DBE: min = 0.5, max = 40.0
Selected filters: None

Monoisotopic Mass, Odd and Even Electron Ions
33 formula(e) evaluated with 1 results within limits (up to 51 closest results for each mass)
Elements Used:
C: 0-200 H: 0-400 O: 8-11

pt20-12 KIB Autospec Premier
15:19:27 25-Aug-2011 M110825EA-17AFAMM 24 (2.203) P776
Voltage EI+ 782.4024 1.60e4

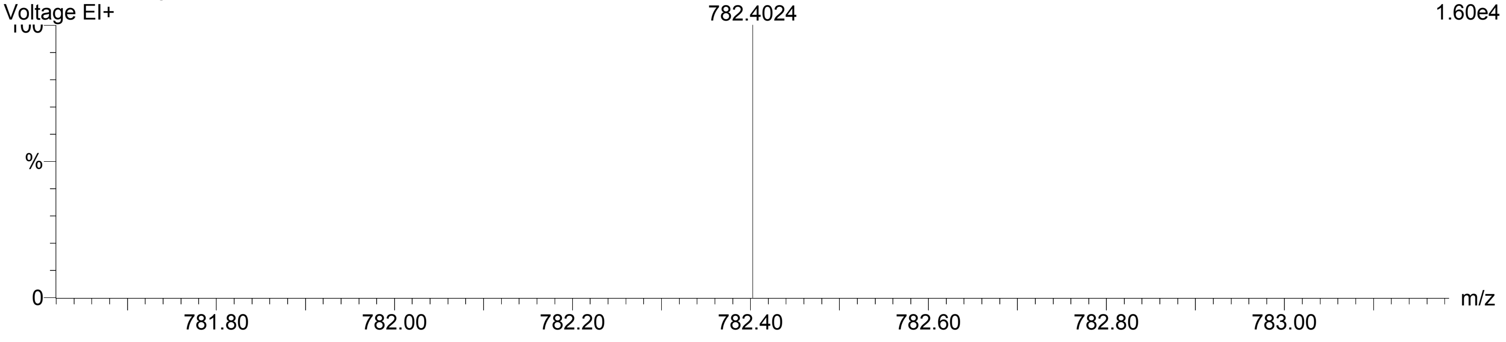

Minimum:				0.5		
Maximum:		100.0	10.0	40.0		
Mass	Calc. Mass	mDa	PPM	DBE	i-FIT	Formula
782.4024	782.4030	-0.6	-0.8	19.0	5554021.5	C47 H58 O10

pt20-7

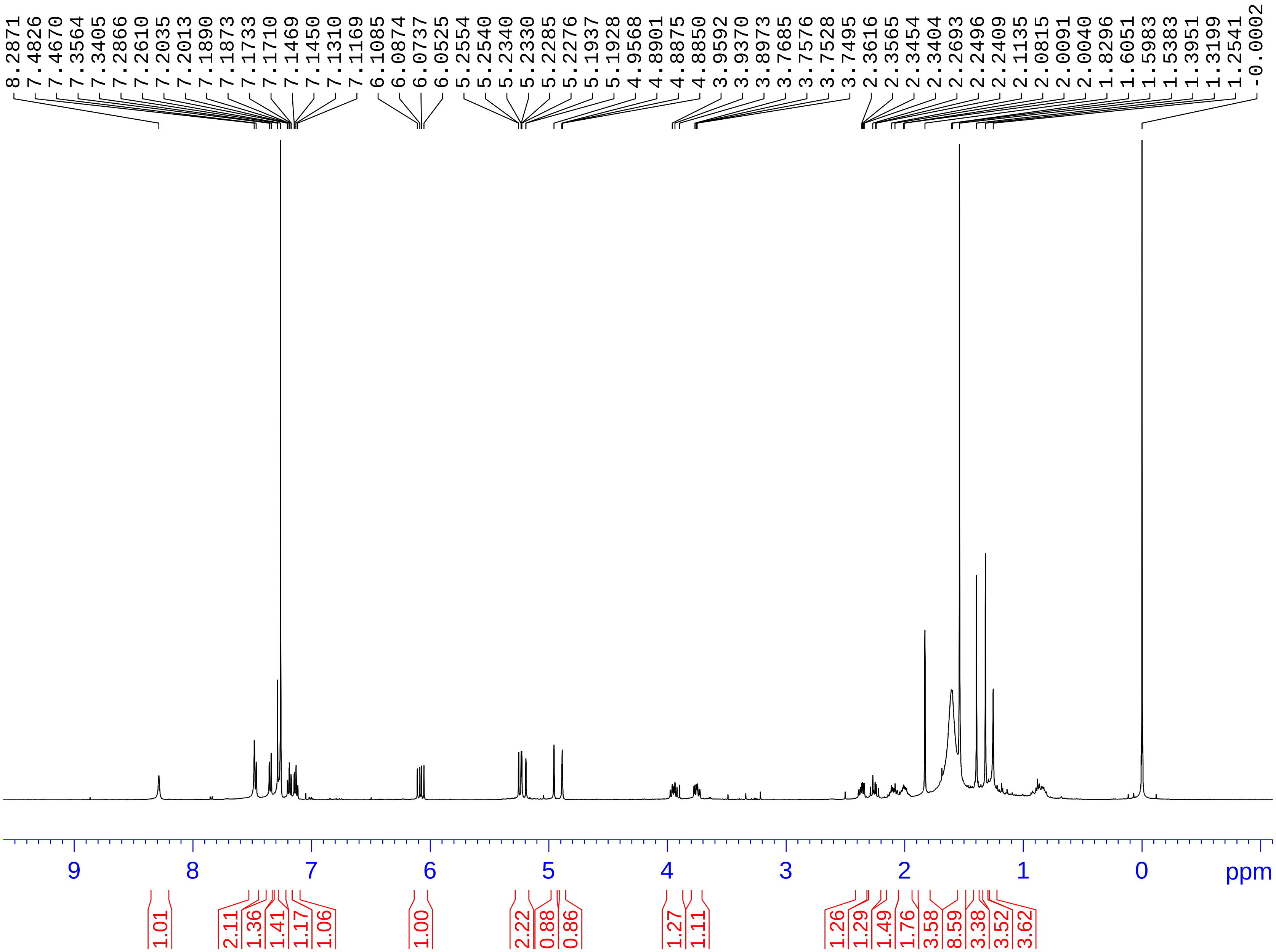

NAME	110628	
EXPNO	11	
PROCNO	1	
Date_	20110701	
Time	16.28	
INSTRUM	spect	
PROBHD	5 mm PABBO BB-	
PULPROG	zg30	
TD	65536	
SOLVENT	CDCl3	
NS	128	
DS	0	
SWH	10330.578	Hz
FIDRES	0.157632	Hz
AQ	3.1719923	sec
RG	161	
DW	48.400	usec
DE	6.50	usec
TE	298.0	K
D1	1.00000000	sec
TD0	1	

======== CHANNEL f1 ========

NUC1	1H	
P1	11.90	usec
PL1	0.35	dB
PL1W	19.76737595	W
SFO1	500.1330885	MHz
SI	32768	
SF	500.1300131	MHz
WDW	EM	
SSB	0	
LB	0.30	Hz
GB	0	
PC	1.00	

pt20-7

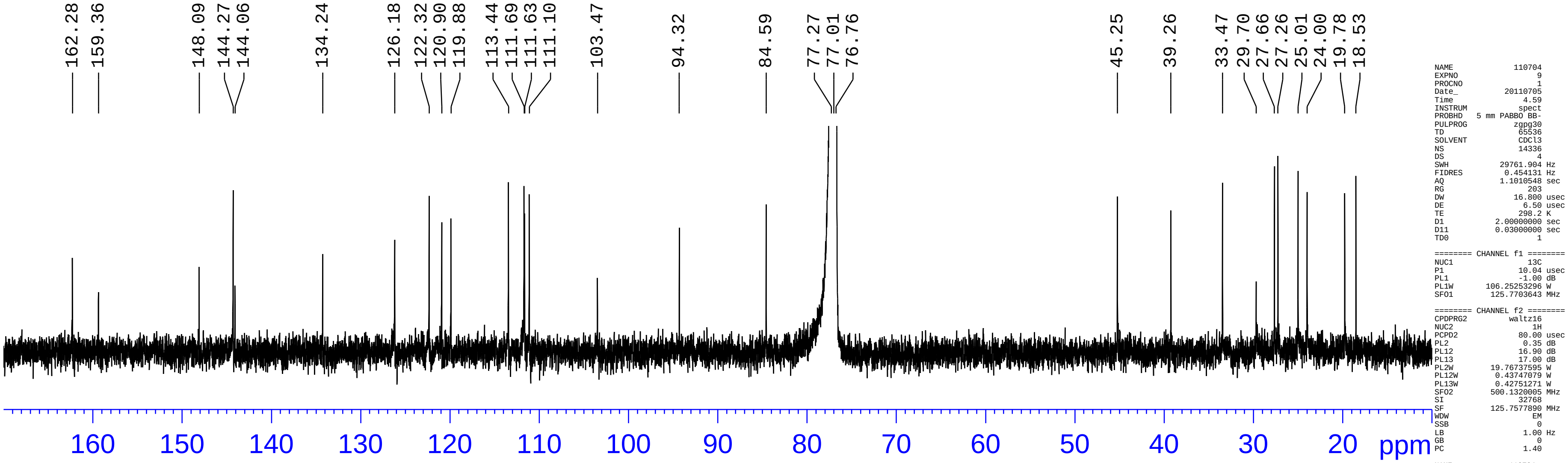

DEPT90

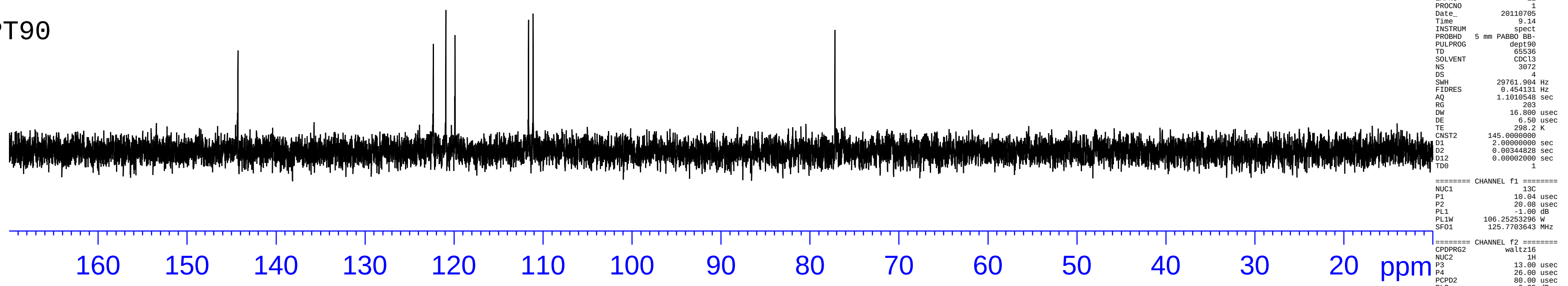

DEPT135

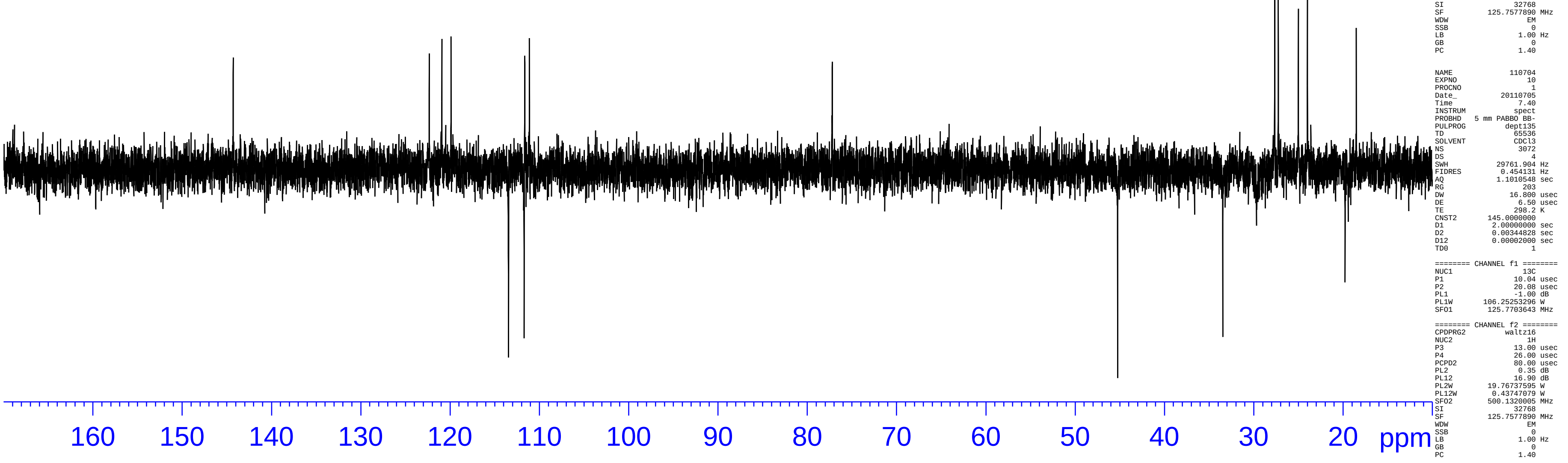

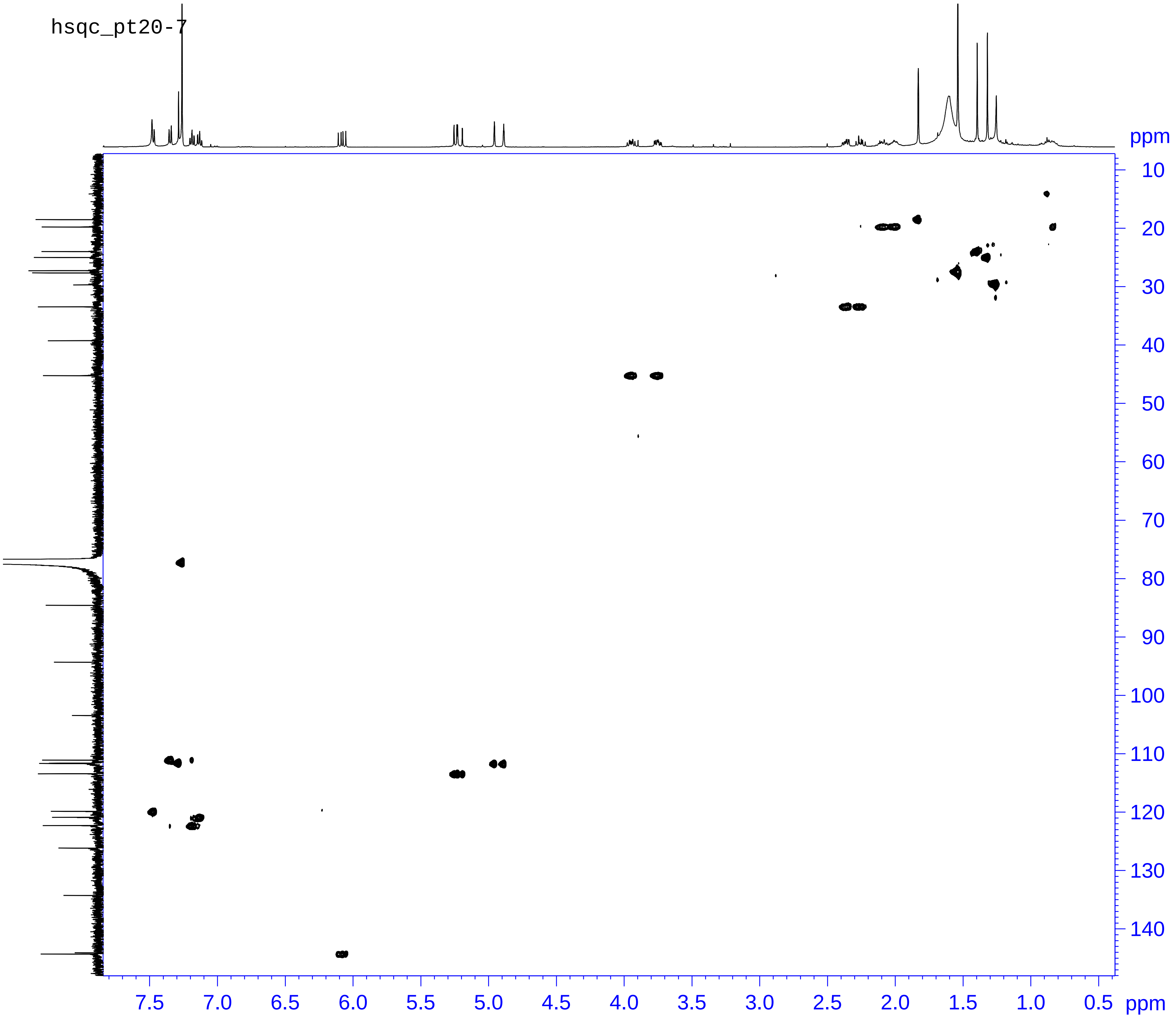


```
NAME              110711
EXPNO                  2
PROCNO                 1
Date_           20110712
Time                8.33
INSTRUM            spect
PROBHD   5 mm PABBO BB-
PULPROG   hsqcetgpsisp2
TD                  2048
SOLVENT            CDCl3
NS                    64
DS                    16
SWH             5000.000 Hz
FIDRES          2.441406 Hz
AQ             0.2048500 sec
RG                   203
DW               100.000 usec
DE                  6.50 usec
TE                 298.3 K
CNST2        145.0000000
D0            0.00000300 sec
D1            2.00000000 sec
D4            0.00172414 sec
D11           0.03000000 sec
D16           0.00020000 sec
D24           0.00086207 sec
IN0           0.00002340 sec
ZGOPTNS

======== CHANNEL f1 ========
NUC1                  1H
P1                 11.90 usec
P2                 23.80 usec
P28                 0.00 usec
PL1                 0.35 dB
PL1W         19.76737595 W
SFO1         500.1322506 MHz

======== CHANNEL f2 ========
CPDPRG2             garp
NUC2                 13C
P3                 11.90 usec
P4                 23.80 usec
P14               500.00 usec
PCPD2              70.00 usec
PL0               120.00 dB
PL2                 1.00 dB
PL12               16.39 dB
PL0W          0.00000000 W
PL2W         67.04081726 W
PL12W         1.93793559 W
SFO2         125.7678496 MHz
SP3                 7.65 dB
SPNAM3    Crp60,0.5,20.1
SPOAL3             0.500
SPOFFS3             0.00 Hz

====== GRADIENT CHANNEL =====
GPNAM1          SINE.100
GPNAM2          SINE.100
GPNAM3          SINE.100
GPNAM4          SINE.100
GPZ1               80.00 %
GPZ2               20.10 %
GPZ3               11.00 %
GPZ4               -5.00 %
P16              1000.00 usec
P19               600.00 usec
ND0                    2
TD                   230
SFO1            125.7678 MHz
FIDRES         92.958847 Hz
SW               170.000 ppm
FnMODE     Echo-Antiecho
SI                  1024
SF           500.1300120 MHz
WDW                QSINE
SSB                    2
LB                  0.00 Hz
GB                     0
PC                  1.40
SI                  1024
MC2        echo-antiecho
SF           125.7577790 MHz
WDW                QSINE
SSB                    2
LB                  0.00 Hz
GB                     0
```

hmbc_pt20-7

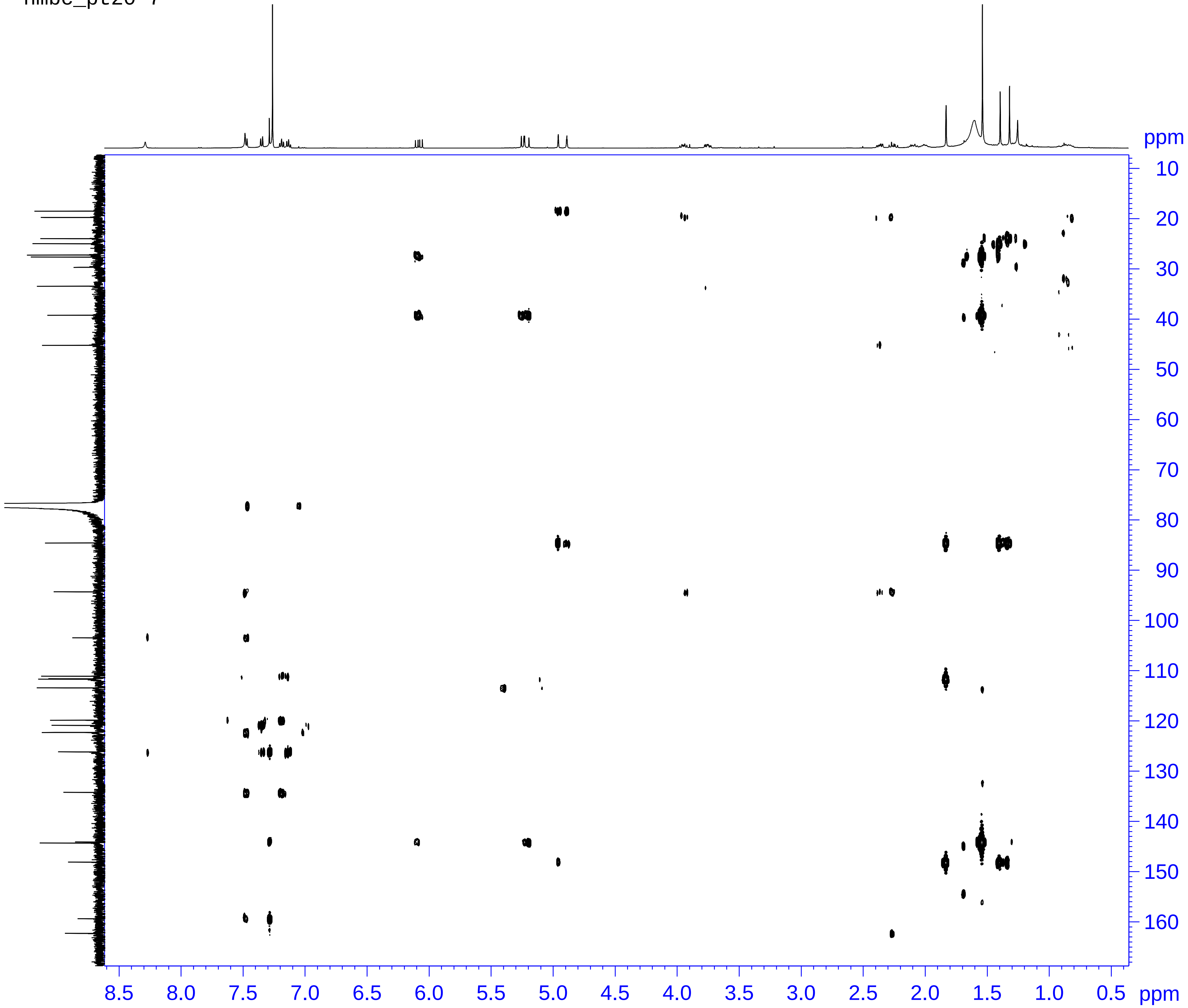


```
NAME             110711
EXPNO                 3
PROCNO                1
Date_          20110711
Time              11.03
INSTRUM           spect
PROBHD   5 mm PABBO BB-
PULPROG    hmbcetgpl2nd
TD                 4096
SOLVENT           CDCl3
NS                  128
DS                   16
SWH            5000.000 Hz
FIDRES         1.220703 Hz
AQ            0.4096500 sec
RG                  203
DW              100.000 usec
DE                 6.50 usec
TE                298.2 K
CNST6       120.0000000
CNST7       180.0000000
CNST13        7.0000000
CNST30        0.5981200
D0           0.00000300 sec
D1           1.50000000 sec
D6           0.07142857 sec
D16          0.00020000 sec
IN0          0.00002210 sec

======== CHANNEL f1 ========
NUC1                 1H
P1                11.90 usec
P2                23.80 usec
PL1                0.35 dB
PL1W        19.76737595 W
SFO1        500.1322506 MHz

======== CHANNEL f2 ========
NUC2                13C
P3                11.90 usec
P24             2000.00 usec
PL2                1.00 dB
PL2W        67.04081726 W
SFO2        125.7684784 MHz
SP7                7.65 dB
SPNAM7      Crp60comp.4
SPOAL7            0.500
SPOFFS7            0.00 Hz

====== GRADIENT CHANNEL ====
GPNAM1         SINE.100
GPNAM3         SINE.100
GPNAM4         SINE.100
GPNAM5         SINE.100
GPZ1              80.00 %
GPZ3              15.00 %
GPZ4             -10.00 %
GPZ5              -5.00 %
P16             1000.00 usec
ND0                   2
TD                  256
SFO1           125.7685 MHz
FIDRES        88.430962 Hz
SW              180.000 ppm
FnMODE     Echo-Antiecho
SI                 1024
SF          500.1300120 MHz
WDW                SINE
SSB                   0
LB                 0.00 Hz
GB                    0
PC                 1.40
SI                 1024
MC2       echo-antiecho
SF          125.7577790 MHz
WDW                SINE
SSB                   0
LB                 0.00 Hz
GB                    0
```

cosy_pt20-7

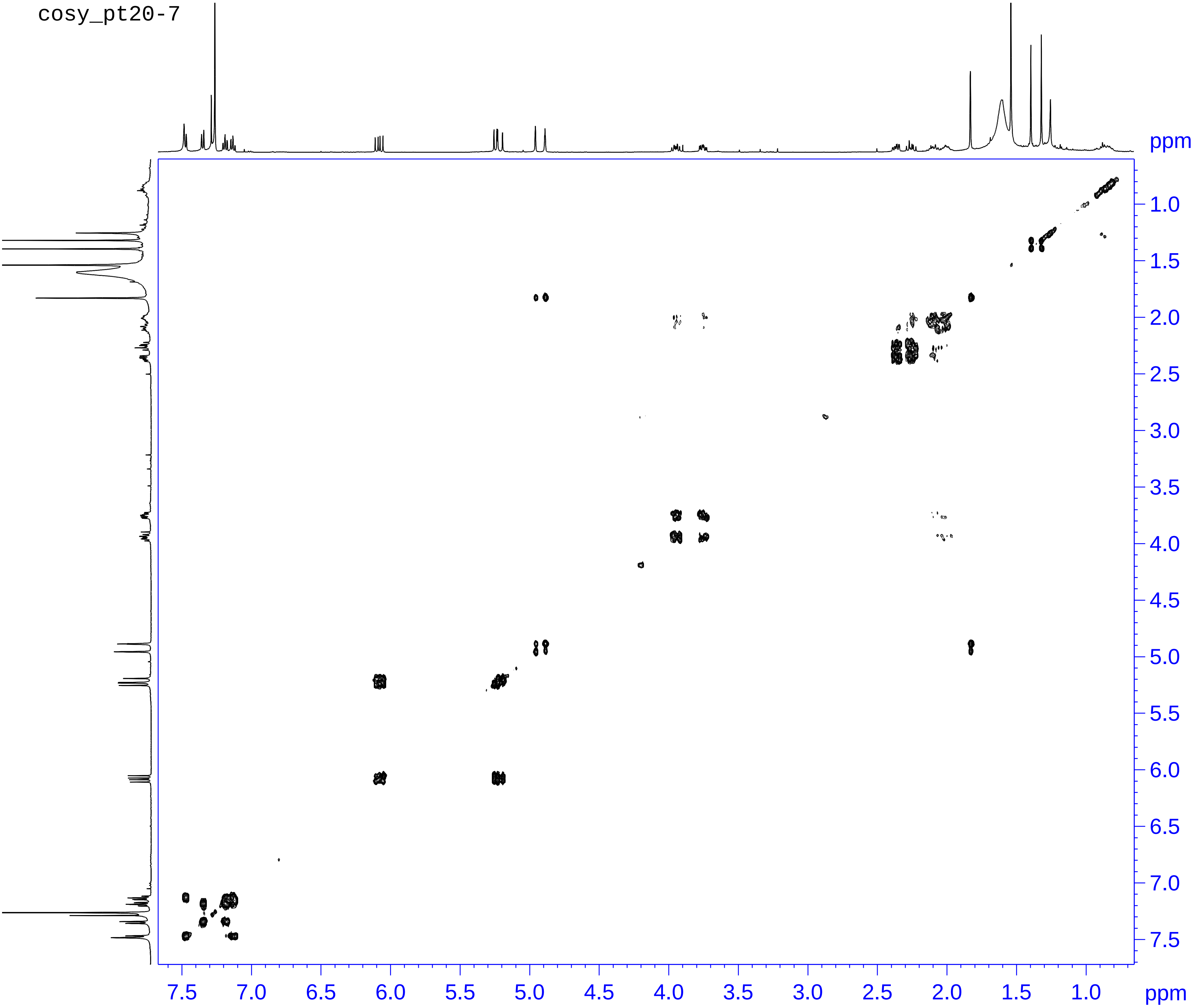


```
NAME                110711
EXPNO                    1
PROCNO                   1
Date_             20110711
Time                  9.41
INSTRUM              spect
PROBHD   5 mm PABBO BB-
PULPROG         cosygpmfqf
TD                    2048
SOLVENT              CDCl3
NS                       8
DS                      16
SWH               5000.000 Hz
FIDRES            2.441406 Hz
AQ               0.2048500 sec
RG                     203
DW                 100.000 usec
DE                    6.50 usec
TE                   298.2 K
D0              0.00000300 sec
D1              2.00000000 sec
D13             0.00000400 sec
D16             0.00020000 sec
IN0             0.00019995 sec

======== CHANNEL f1 ========
NUC1                    1H
P1                   11.90 usec
PL1                   0.35 dB
PL1W           19.76737595 W
SFO1           500.1322506 MHz

====== GRADIENT CHANNEL =====
GPNAM1           SINE.100
GPNAM2           SINE.100
GPNAM3           SINE.100
GPZ1                16.00 %
GPZ2                12.00 %
GPZ3                40.00 %
P16               1000.00 usec
ND0                     1
TD                    256
SFO1             500.1323 MHz
FIDRES          19.536415 Hz
SW                 10.000 ppm
FnMODE                 QF
SI                   1024
SF            500.1300129 MHz
WDW                  SINE
SSB                     0
LB                   0.00 Hz
GB                      0
PC                   1.40
SI                   1024
MC2                    QF
SF            500.1300129 MHz
WDW                  SINE
SSB                     0
LB                   0.00 Hz
GB                      0
```

noesy_pt20-7

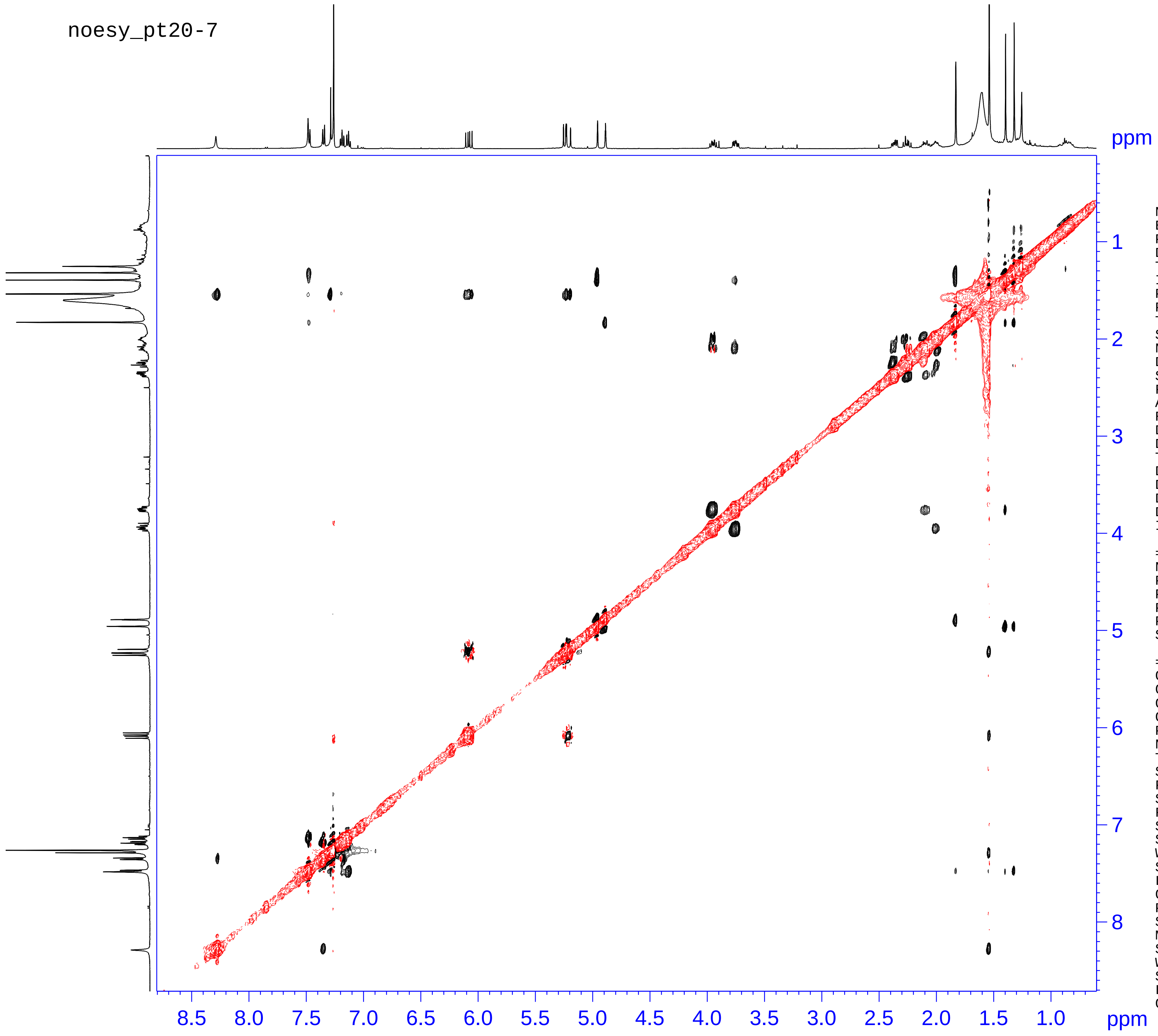

NAME	110711
EXPNO	4
PROCNO	1
Date_	20110712
Time	5.11
INSTRUM	spect
PROBHD	5 mm PABBO BB-
PULPROG	noesygpph
TD	2048
SOLVENT	CDCl3
NS	16
DS	8
SWH	5000.000 Hz
FIDRES	2.441406 Hz
AQ	0.2048500 sec
RG	203
DW	100.000 usec
DE	6.50 usec
TE	298.2 K
D0	0.00008482 sec
D1	2.00000000 sec
D8	0.69999999 sec
D16	0.00020000 sec
IN0	0.00019995 sec

======== CHANNEL f1 ========

NUC1	1H
P1	11.90 usec
P2	23.80 usec
PL1	0.35 dB
PL1W	19.76737595 W
SFO1	500.1322506 MHz

====== GRADIENT CHANNEL =====

GPNAM1	SINE.100
GPNAM2	SINE.100
GPZ1	40.00 %
GPZ2	-40.00 %
P16	1000.00 usec
ND0	1
TD	256
SFO1	500.1323 MHz
FIDRES	19.536415 Hz
SW	10.000 ppm
FnMODE	States-TPPI
SI	1024
SF	500.1300120 MHz
WDW	QSINE
SSB	2
LB	0.00 Hz
GB	0
PC	1.00
SI	1024
MC2	States-TPPI
SF	500.1300120 MHz
WDW	QSINE
SSB	2
LB	0.00 Hz
GB	0

Single Mass Analysis (displaying only valid results)

Tolerance = 10.0 PPM / DBE: min = 0.5, max = 40.0
Selected filters: None

Monoisotopic Mass, Odd and Even Electron Ions
17 formula(e) evaluated with 1 results within limits (up to 51 closest results for each mass)
Elements Used:
C: 0-200 H: 0-400 N: 3-3 O: 1-3

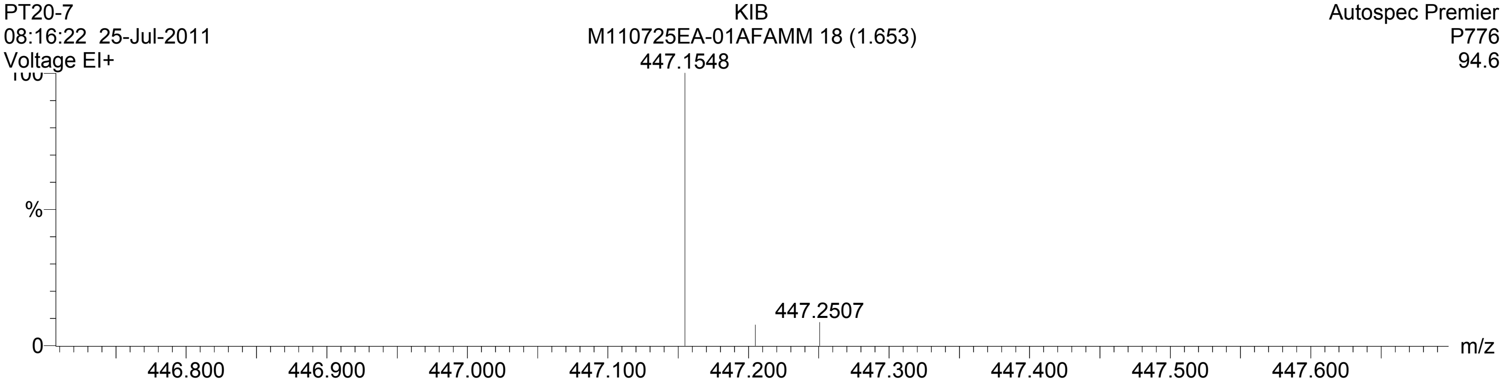

Minimum:				0.5		
Maximum:		100.0	10.0	40.0		
Mass	Calc. Mass	mDa	PPM	DBE	i-FIT	Formula
447.2507	447.2522	-1.5	-3.4	13.0	5546026.0	C27 H33 N3 O3
